# Efficacy of Isomaltulose Compared to Sucrose in Modulating Endothelial Function in Overweight Adults

**DOI:** 10.3390/nu12010141

**Published:** 2020-01-03

**Authors:** Eric de Groot, Lisa Schweitzer, Stephan Theis

**Affiliations:** 1 Imagelabonline & Cardiovascular, 4117 GV Erichem, The Netherlands; 2 Amsterdam UMC—Location Academic Medical Centre, Departments of Clinical Epidemiology, Biostatistics and Bioinformatics, and Gastroenterology, 1105 AZ Amsterdam, The Netherlands; 3 BENEO-Institute, c/o BENEO GmbH, Wormser Straße 11, 67283 Obrigheim, Pfalz, Germany; lisa.schweitzer@beneo.com (L.S.); stephan.theis@beneo.com (S.T.)

**Keywords:** isomaltulose, endothelial function, glycemic index, carbohydrates, insulin resistance, cardiovascular health

## Abstract

Hyperglycemia is linked to impaired arterial endothelial function (EF), an early sign of cardiovascular disease. We compared the efficacy of low-glycemic index isomaltulose (Palatinose™) with that of sucrose in modulating EF, as assessed by flow-mediated dilation (FMD). In this double-blinded cross-over study, 80 overweight mildly hypertensive subjects were randomized to receive 50 g of either isomaltulose or sucrose. On two non-consecutive days, brachial artery ultrasound FMD scans were obtained prior to and hourly (T0–T3) after carbohydrate load. Blood was drawn immediately after scanning. Glucose and insulin levels were analyzed. Overall, the FMD decrease was attenuated by isomaltulose compared to sucrose (ΔFMD = −0.003% and −0.151%; *p* > 0.05 for the interaction treatment x period). At T2, FMD was significantly higher after isomaltulose administration compared to that after sucrose administration (FMD = 5.9 ± 2.9% and 5.4 ± 2.6%, *p* = 0.047). Pearson correlations between FMD and blood glucose showed a trend for a negative association at T0 and T2 independently of the carbohydrate (r-range = −0.20 to −0.23, *p* < 0.1). Sub-analysis suggested a lower FMD in insulin-resistant (IR) compared to insulin-sensitive subjects. Isomaltulose attenuated the postprandial decline of FMD, particularly in IR persons. These data support the potential of isomaltulose to preserve the endothelial function postprandially and consequently play a favorable role in cardiovascular health.

## 1. Introduction

Endothelial function (EF) plays a pivotal role in cardiovascular health. Impaired EF is a prominent health concern, as it typically contributes to cardiovascular disease (CVD), i.e., hypertension, atherosclerosis, or myocardial infarction [1,2]. In an aging population, the high prevalence, still increasing incidence, and alarming number of deaths due to CVD every year worldwide [3,4,5] demonstrate the importance of counterbalancing CVD with the development of innovative preventive strategies integrated in everyday life.

EF can be assessed by brachial artery ultrasound imaging. With this completely non-invasive, subject-friendly technique, the shear stress-induced flow-mediated endothelium-dependent vasodilation (FMD) of the brachial artery can be measured [6]. A high FMD (e.g., 10% and above) corresponds to a vital endothelium; a low or no FMD (e.g., 5 to 0%) indicates impaired EF or endothelial dysfunction (ED). Brachial ultrasound FMD measurements are well reproducible and are considered a validated early marker of CVD risk [7]. Although the underlying mechanism of ED is not yet completely understood, a key vasodilator is known to be nitric oxide (NO). A decline in NO synthesis, through the inhibition of, e.g., NO synthase, leads to an impaired vasodilation of blood vessels [8], which contributes to hypertension and promotes the clinical symptoms of CVD. Besides a reduced NO bioavailability, ED is characterized by oxidative stress and an increase of inflammatory as well as thrombotic factors [9]. Furthermore, it is well known that clinical states of insulin resistance, including obesity, metabolic syndrome, and diabetes mellitus, are associated with impaired endothelium-dependent vasodilation [9,10]. This can be explained by the vasodilatory properties of insulin, mediating NO synthesis via its stimulation of the insulin receptor and, finally, increasing NO bioavailability in healthy humans [8]. In contrast, in insulin-resistant persons, insulin signaling is impaired not only at the metabolic but also at the vascular level [11], and moreover, the concentration of the vasoconstrictor endothelin-1 was found to be increased [10]. Thus, insulin resistance is accompanied by an increased risk for ED [9].

Besides insulin resistance itself, accumulating evidence indicates a link between EF and postprandial dysmetabolism such as hyperglycemia and its consequences, i.e., vascular events [12,13,14]. Hyperglycemia is postulated to induce oxidative stress and impair EF [15], which is in accordance with the FMD decline occurring directly after food intake [16,17,18,19]. There is some evidence that low-glycemic index (GI) foods may favorably influence EF [20,21]. This was firstly examined by Lavi et al., who investigated differences in postprandial FMD in response to foods with various glycemic indices [21]. The authors found a significant decrease in FMD compared to baseline two hours after food ingestion, which was the highest after glucose ingestion, followed by cornflakes intake. Although a low-GI food (fiber-rich cereals) showed a slight decrease in FMD in the postprandial state as well, the decrease was not significant and comparable with the FMD decrease induced by water [21]. In alignment with the results of Lavi et al., other dietary intervention studies previously showed an improvement in blood pressure or pulse wave velocity due to low-GI compared to high-GI diets [22,23], which would also explain the positive association between glycemic index and CVD [24,25,26].

The hypothesis that hyperglycemia causes ED is supported by the observation that overweight and obese individuals and those with diabetes, either type 1 or type 2, are more prone to ED [8,11,27,28]. As these persons are at higher risk of postprandial dysmetabolism, continuous exposure to elevated blood glucose induces oxidative stress and chronic inflammation [18,29], both predictors of ED. Moreover, insulin resistance might have additive adverse effects on the vascular function [11].

On the basis of this knowledge and considering the large intake of high-GI foods that characterizes today’s Western diet, low-GI foods might be effective in preventing ED. For instance, a substantial replacement of sugar by low-GI carbohydrates, e.g., isomaltulose (Palatinose™, BENEO GmbH, 68165 Mannheim, Germany), may contribute to improved cardiovascular health, as indicated by Keller et al. [30]. During this study on inactivity in healthy young men, prolonged vascular relaxation, assessed by the augmentation index, was observed after isomaltulose ingestion compared to sucrose administration [30].

Both sucrose and isomaltulose are disaccharides consisting of a glucose and a fructose monomer, which provide the same amount of calories. However, they differ in their glycosidic bond, which is α-1,2 for sucrose and α-1,6 for isomaltulose [31]. The bond present in isomaltulose causes its slower absorption in the small intestine, thus resulting in a low GI (32 for isomaltulose vs. 65 for sucrose) [32,33].

The present exploratory study aimed to investigate the acute effects of isomaltulose versus those of sucrose on endothelium-dependent vasodilation, i.e., FMD, in overweight/obese subjects. The lower blood glucose response following isomaltulose load may allow for enhanced preservation of EF. We therefore hypothesized that, compared to sucrose, isomaltulose would cause lesser postprandial impairment of EF in overweight/obese individuals.

## 2. Materials and Methods

### 2.1. Subjects

Between August 2016 and March 2017, overweight and obese subjects with mild hypertension (systolic/diastolic blood pressure: 130–159/85–99 mmHg) were recruited by Atlantia Food Clinical Trials CRO (Cork, Ireland). After screening, healthy persons aged 25–60 years were included if their body weight was stable (≤5% change over the past 3 months) and their physical activity level was low to moderate (assessed by the International Physical Activity Questionnaire-Short form). Exclusion criteria were use of medications influencing glucose metabolism (e.g., antidiabetics) or EF (e.g., anti-hypertensive and anti-atherosclerotic drugs) and chronic or acute diseases. Smokers, heavy coffee drinkers, and pregnant women were further excluded. Throughout the study, subjects were asked to follow their usual diet and exercise routine and to avoid the named medications that could interfere with the study outcomes.

The study protocol was approved by the Clinical Research Ethics Committee of the Cork Teaching Hospitals. All participants gave their written informed consent before the study’s start according to the ICH Guidelines on Good Clinical Practice and the declaration of Helsinki. The trial was registered at clinicaltrials.gov (NCT03986775).

### 2.2. Study Protocol

**Figure 1:**
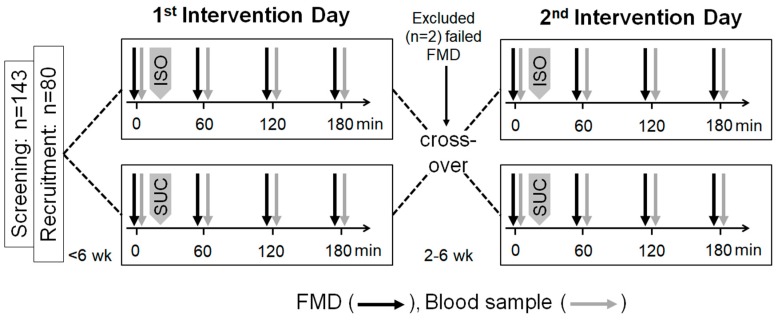
Outline of the study protocol. FMD, flow-mediated dilation; ISO, citrus drink containing 50 g of isomaltulose; SUC, citrus drink containing 50 g of sucrose.

An outline of the study protocol is depicted in Figure 1. Beside the screening visit, in this double-blinded controlled cross-over trial, participants were tested on two different intervention days, which were separated by a 2–6-week wash-out period. On the two intervention days, the participants attended the clinic after an overnight fast of at least 10 h. To minimize confounding variables, for premenopausal women, the intervention days were within the first seven days of their menstrual cycle. Moreover, the subjects were asked to avoid high-fat and high-flavonoid foods (e.g., blueberries, dark chocolate, etc.) for 24 h and caffeine for 10 h and to abstain from exercise for 12 h before the intervention.

The order of the assessments was exactly the same on both intervention days. Prior to carbohydrate load (T0), a brachial ultrasound FMD scan was performed, with blood drawn shortly thereafter. All subjects were blinded to treatment and randomly assigned to consume either 50 g of isomaltulose (Palatinose™, provided by BENEO GmbH, Mannheim, Germany) or 50 g sucrose in the form of instant citrus drinks. Therefore, drink powders were dissolved in 500 mL of water shortly before consumption. The subjects were instructed to consume the citrus drink within 10 min after the T0 FMD. At 60 min (T1), 120 min (T2), and 180 min (T3) after the start of the citrus drink consumption, FMD scans and blood sample collections were performed.

### 2.3. Flow-Mediated Dilation

Ultrasound FMD scanning procedures were strictly standardized and quality-controlled as described in extenso elsewhere [34,35]. In short, prior to the start of the study, two research technicians on site (Atlantia Food Clinical Trials CRO, Cork, Ireland) were trained and certified according to pre-set criteria of the FMD core lab (Imagelabonline & Cardiovascular, Erichem, The Netherlands). All scans were performed with an Ultrasonix Sonix SP ultrasound instrument equipped with an L14-5MHz vascular transducer and fixed presets throughout the study (Analogic Corporation, Peabody, MD, USA).

The examinations took place in a quiet room at temperatures between 20 °C and 26 °C and dimmed lighting. For each subject on both test days, initial and follow-up scans were performed by the same technician. Subjects comfortably reclined for 20 min prior to scans. During visits, the subjects’ right arm rested in the arm-supporting cups of the probe-holder arm rest in a fixed position during the 9′ scan procedures. The brachial artery was scanned longitudinally to obtain high-quality reproducible scans of the arterial lumen with the ultrasound probe 5–10 cm proximal to the antecubital fossa, its position documented with a tape measure for accurate repositioning of the probe in the second visit. On the lower arm, a blood pressure cuff was inflated 40 to 50 mmHg above systolic pressure for 5 min to temporarily occlude the brachial artery. According to a fixed ultrasound imaging application protocol, 1 min prior and 3 min after arterial occlusion, electrocardiogram (EKG)-triggered images were recorded in DICOM clips, with each full-frame image in the clip to provide a lumen diameter measurement. For off-line scan analyses, the clips were directly and securely transferred from the ultrasound equipment to the core lab (Imagelabonline & Cardiovascular, Erichem, The Netherlands) for immediate in-study quality control and investigational site feedback by the core lab FMD experts. For the treatment efficacy image analyses, per-subject batches of FMD scans were provided to the trained and certified technicians of the core lab. Technicians were blinded to subject demographic, clinical, and treatment data, as well as scan visit order. Validated and FDA-approved image analysis software was used (Brachial Analyzer, Medical Imaging Applications, Coralville, IA, USA).

FMD was calculated as the percentage of change in the brachial arterial diameter:

(1) $$\mathrm{Brachial}\;\mathrm{FMD}\;\left(\%\right)=\frac{\mathrm{Maximum}\;\mathrm{diameter}\;\left(\mathrm{mm}\right)-\mathrm{Baseline}\;\mathrm{diameter}\;\left(\mathrm{mm}\right)}{\mathrm{Baseline}\;\mathrm{diameter}\;\left(\mathrm{mm}\right)}\times 100$$

where the brachial arterial lumen diameter at ‘baseline’ was the average during 1 min prior to cuff-occlusion recording of the brachial artery diameter, and the maximum brachial arterial lumen diameter was the maximum diameter during the 3 min of post-cuff release recording.

### 2.4. Blood Sampling and Analytical Methods

A vein cannula was inserted into the subject’s left arm, and a blood sample (12 mL) was collected shortly after FMD measurement at T0, T1, T2, and T3 during both intervention days. Serum glucose concentrations were measured using the hexokinase method (3L82, ARCHITECT c system, Abbott Laboratories, Abbott Park, IL 60064, USA). Serum insulin levels were determined using a chemiluminescent microparticle immunoassay (8K41, ARCHITECT c system, Abbott Laboratories, USA). Homeostasis model assessment (HOMA) index was calculated using the following equation: fasting glucose (mmol/L) × fasting insulin (µU/L)/22.5.

### 2.5. Statistics

Statistical analyses were carried out using SPSS Version 24 and SAS Version 9.3. Sample size was calculated based on primary outcome FMD. On the basis of intra-sonographer repeatability of 1.25%FMD, α of 0.05 (two-sided), and β of 0.1 and on an effect size of 0.9%FMD, the minimal required subject number was 68. To account for drop outs and FMD assessment failures, in total 80 subjects were included.

Descriptive characteristics are presented as mean ± SD. As the data were normally distributed (Kolmogorov–Smirnov test, *p* > 0.05), parametric tests were used. To compare the baseline characteristics of the isomaltulose-first and sucrose-first groups, unpaired *t* test for continuous variables and χ^2^ test for categorical variables were applied.

A mixed repeated-measures analysis of variance (MMRM) with terms for treatment and period was performed to observe differences in endothelial responses following investigational products adjusted for baseline FMD. In this primary statistical analysis, only FMD data of those subjects with fully completed datasets of the four FMD measurements at visit 1 and the 4 FMD measurements at visit 2 were included.

To compare differences in FMD, blood glucose, and insulin levels for the carbohydrates, an exploratory analysis at each of the time points within subjects between visits was performed using the paired *t* test. In these statistical analyses, subjects were included provided that both FMD measurements at a given time point were available. Pearson correlations were carried out to detect associations between FMD and blood glucose as well as insulin levels at different time points.

For the evaluation of differences in the endothelial response depending on the insulin sensitivity status, subjects were classified according to their HOMA index on their first intervention day, and a subgroup analysis was performed. Subjects with HOMA < 2.5 were considered to be insulin-sensitive (IS), whereas subjects with HOMA ≥ 2.5 were considered as insulin-resistant (IR). To see differences in the change of FMD from baseline (∆FMD_Tx-T0_) between IS and IR subjects, unpaired *t* test was used. Two-sided statistics was performed with the significance level set at *p* < 0.05.

## 3. Results

### 3.1. Descriptive Characteristics

In total, 143 volunteers were screened for eligibility, of whom 80 were randomized, as all inclusion criteria were met. In total, 78 subjects completed the study, 39 for each randomization sequence. Two subjects dropped out due to anatomical difficulty in obtaining a proper FMD measurement at baseline.

The baseline characteristics are presented in Table 1. Overall, age ranged from 28 to 60 years, and body mass index (BMI) from 24.8 to 34.9 kg/m². With regard to BMI, blood pressure, FMD, and blood glucose and insulin levels, sequence groups were comparable. In the isomaltulose-first and sucrose-first groups, 10 and 14 subjects (25% and 35%), respectively, were past smokers, without a statistically significant difference (*p* = 0.33). None of the subjects had a high level of physical activity; 47 subjects (58.8%) had a moderate level and 33 (41.3%) a low level of physical activity. This was not different between the randomization sequences (*p* = 0.26).

### 3.2. Effects of Acute Isomaltulose and Sucrose Intakes on Endothelial Function

Of the 80 subjects, 78 were subjected to the four brachial ultrasound FMD scans at each of the two visits. This amounted to 624 scans, of which 596 provided FMD data (a success rate of 95.5%). For 19 of the subjects, due to anatomic and/or ultrasound imaging reasons, 28 of the brachial scans could not provide FMD data (4.5%).

For the primary statistical analysis, the available fully completed FMD datasets of 61 subjects were used. The overall FMD change from baseline showed maintenance of the endothelial function after isomaltulose administration (ΔFMD_ISO,T1–T3_ = −0.003%) and a minor, non-significant, negative change after sucrose administration (ΔFMD_SUC,T1–T3_ = −0.151%). The mixed repeated-measures analysis of variance controlling for baseline FMD showed no significant interaction between treatment and period (*p* > 0.05).

**Figure 2:**
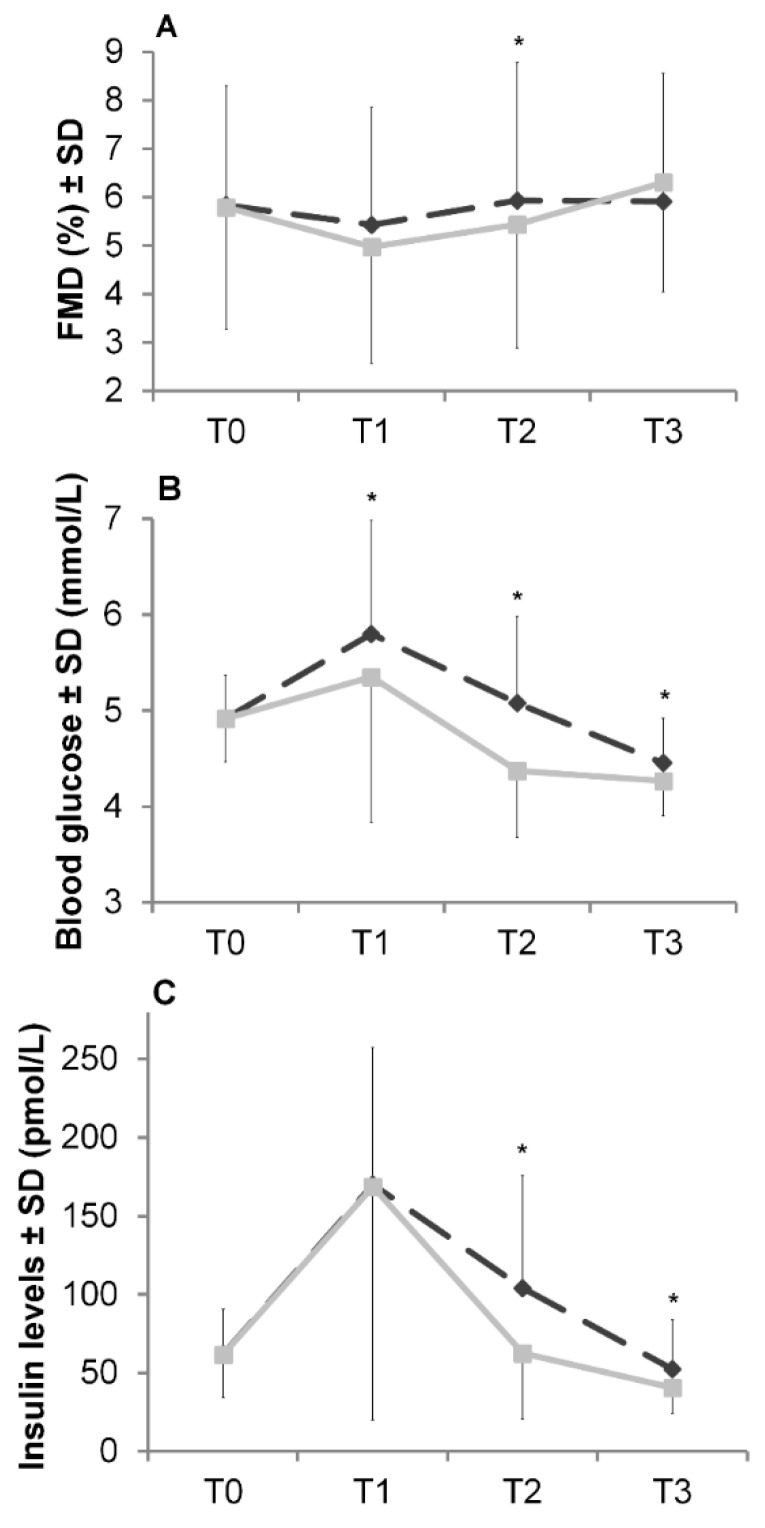
Basal (T0) and postprandial (T1–T3) FMD (**A**), blood glucose (**B**), and insulin levels (**C**) following the ingestion of isomaltulose (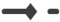) and sucrose (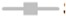). All values are reported as mean ± SD; T0 = 0 min, T1 = 60 min, T2 = 120 min, T3 = 180 min; * Significant difference between isomaltulose and sucrose; paired *t* test: *p* < 0.05.

The consumption of 50 g of carbohydrate led to a decrease in FMD at T1, with changes from baseline FMD (ΔFMD) of −0.40% and −0.74% for isomaltulose (ΔFMD_ISO,T1_) and sucrose (ΔFMD_SUC,T1_), respectively (see Figure 2A). At T2, FMD increased and, after isomaltulose intake, reached baseline FMD. In contrast, after sucrose intake, although there was an increase from T1 to T2, FMD at T2 was still below baseline FMD (ΔFMD_SUC,T2_ = −0.27%, ΔFMD_ISO,T2_ = +0.12%).

In further explorative analyses, between-visit, within-subject paired FMD data points of the four brachial ultrasound scan time points T0, T1, T2, and T3 h were available in 73 (94%), 71 (91%), 72 (92%), and 71 (91%) subjects, respectively. After two hours (T2), the absolute FMD was significantly higher following isomaltulose intake compared to sucrose intake (at T2) (FMD_ISO,T2_ = 5.9 ± 2.9%, FMD_SUC,T2_ = 5.4 ± 2.6%, paired *t* test: *p* < 0.05). When compared to T2, FMD following isomaltulose administration was almost unchanged at T3, with a change from baseline FMD of +0.19%. In contrast, for sucrose, a rebound was observed at T3, resulting in +0.58% compared to baseline FMD.

### 3.3. Blood Analyses and Associations between Blood Glucose and Insulin Levels with Endothelial Function

As expected, there was a rise in blood glucose and insulin levels after carbohydrate ingestion (Figure 2B,C). With regard to the blood glucose response, an exploratory analysis revealed significantly higher blood glucose levels at all time points after isomaltulose ingestion compared to sucrose ingestion (paired *t* test: all *p* values <0.05). For insulin, the response levels were significantly higher after isomaltulose administration compared to sucrose intake (paired *t* test: *p* < 0.05), with the exception of T1.

Independent of the carbohydrate, a correlation analysis between FMD and blood glucose levels at different time points showed a trend for a negative association at T0 and T2 (r-range = −0.202 to −0.230, *p* < 0.1; data not shown). With respect to insulin response, no associations with baseline or postprandial FMD were observed.

### 3.4. Differences in Endothelial Function Depending on Insulin Sensitivity Status

For this exploratory analysis, fasting blood glucose and insulin levels were available for 70 subjects. On the basis of their HOMA index prior to the first intervention, 79% of the subjects were classified as IS, and 21% as IR. Table 2 demonstrates the change in FMD from baseline (∆FMD_Tx-T0_) for IR and IS subjects. Generally, subjects with IR showed a higher decline in FMD at T1 and T2 compared to IS subjects, which was independent of the carbohydrate ingested (unpaired *t* test; *p* > 0.05). When compared to sucrose, the ingestion of isomaltulose showed a lower decline in postprandial FMD at T1 and T2 for both IR and IS subjects (paired *t* test; *p* > 0.05). At T3, the consumption of isomaltulose resulted in an FMD which was comparable to baseline FMD for both IS and IR subjects. In contrast, the consumption of sucrose showed an increase in FMD exceeding the baseline value.

## 4. Discussion

We hypothesized that the decrease in endothelial function caused by food intake could be attenuated by low-GI foods compared to high-GI carbohydrates. The assumption was tested in mildly hypertensive overweight/obese adults using either sucrose (GI = 65) or isomaltulose (GI = 32).

As a novel key finding, our data revealed a preservation of postprandial FMD following the intake of isomaltulose compared to sucrose. Consequently, our results support the hypothesis. The exploratory analysis showed that after two hours, endothelium-dependent vasodilation was significantly higher for isomaltulose ingestion compared to sucrose ingestion (FMD = 5.9 ± 2.9% vs. 5.4 ± 2.6%; paired *t* test: *p* < 0.05, Figure 2A). This finding is in accordance with the literature [20,21]. Lavi et al. investigated the postprandial effects of foods varying in their GI on EF [21]. The higher decrease in FMD after ingestion of high-GI foods found by the authors was confirmed by another intervention study testing different types of rice [20].

Our present study also addresses more general issues regarding high-GI food intake and its physiological consequences. In particular, there is increasing evidence suggesting an association between the GI of foods and CVD [20,25,36,37].

In that context, it is known that postprandial hyperglycemia induces oxidative stress, triggering atherogenic alterations, like secretion of pro-inflammatory cytokines, adhesion molecules, or vasoconstrictive substances (e.g., endothelin-1) [14,38,39]. Consecutive atherogenic alterations contribute to cell damage, including to cells of the vascular endothelium, and when recurring, resulting in ED [16,18,40]. Moreover, ED was observed to be improved through the administration of antioxidants in vitro [41] and in vivo [42]. Previous studies confirmed a lower blood glucose response in the early postprandial state followed by a more sustained glucose response in the later postprandial state with isomaltulose ingestion when compared with ingestion of rapidly absorbable carbohydrates [43,44]. By preventing hyperglycemia, isomaltulose may have less impact on EF and, hence, could be beneficial also for cardiovascular health. In the current study, we did not observe a lower blood glucose response with isomaltulose, which can be attributed to the restrictive blood sampling time points (i.e., only after 1, 2, and 3 h). Rather, the later postprandial state was examined, and early postprandial glucose peaks were probably missed. This is supported by previous studies showing a rapid blood glucose decrease with sucrose ingestion already before and around 60 min [44].

A second explanation for the link between the GI of foods and CVD involves the action of insulin. Because insulin is a vasodilatory agent, insulin resistance might impair EF. Insulin signaling is important for vasodilation through the activation of endothelial NO synthase. Hence, insulin resistance is accompanied by diminished vasodilatory function due to reduced NO bioavailability [9]. Additionally, insulin resistance is associated with increased expression of endothelin-1 [45], which worsens ED by mediating vasoconstriction. Besides lower glycemia, slowly absorbable carbohydrates prevent an excessive postprandial insulin response, particularly in the early postprandial phase, although we could not document this event due to the late timing of blood sampling [31,44,46]. Moreover, a low-GI beverage containing isomaltulose compared to a high-GI beverage led to reduced daylong insulinemia and attenuated insulin resistance after one week of inactivity [30]. Besides metabolic impairment, insulin resistance is associated with vascular resistance [39,47]. Thus, replacing high-GI carbohydrates might positively impact EF through improved insulin sensitivity which, in turn, enhances the vasodilatory effect of insulin. This would be in line with a study by Hurwitz et al. who investigated differences in the endothelial response between IR and IS subjects following the ingestion of high- versus low-carbohydrate meals [48]. In that study, IR subjects, compared to IS subjects, showed a significantly lower FMD already at baseline. Moreover, the authors observed a decline in postprandial FMD following both meals. Independent of the insulin sensitivity status, the decline in postprandial FMD was more pronounced following the high-carbohydrate meal. Also in the present study, we observed a more pronounced decline in postprandial FMD following sucrose intake compared to isomaltulose intake. This was seen in both IS and IR subjects. Yet, data in IR subjects demonstrate a very pronounced FMD drop following sucrose intake, which appeared to be mitigated by isomaltulose. The present data thus indicate that IR persons, i.e., persons whose glucose metabolism is disturbed, could particularly profit from low-GI carbohydrates.

Thirdly, the beneficial effects of GLP-1 on the vascular system in relation to isomaltulose need to be addressed. Due to its slow hydrolysis, isomaltulose enhances GLP-1 secretion compared to rapidly available carbohydrates in both healthy persons [30,33] and diabetics [49]. GLP-1 is known to improve insulin sensitivity as well as secretion and, furthermore, some data suggest that GLP-1 ameliorates EF [50,51]. The insulin-sensitizing and blood glucose-lowering effects of GLP-1 presumably prevent postprandial oxidative stress and thus could contribute to a mitigation of ED.

### Strengths and Limitations

To our knowledge, the present study is the first comparing FMD following the ingestion of sucrose and low-GI isomaltulose. Considering the fact that today’s diet contains high amounts of high-GI foods, which leads to adverse health effects, the present results are of clinical relevance.

Nevertheless, the efficacy study was based on the primary outcome FMD. Hence, blood analysis was limited with regard to the number of time points and intervals chosen, i.e., every 60 min. This most likely contributed to the failure to detect actual blood glucose peaks which are known to occur in the early postprandial phase. The inclusion of further time points in blood analyses and FMD scanning might have provided additional mechanistic insight on the potential associations between blood glucose and insulin levels with EF. Another limitation of this study is the sample size of the subgroup analysis. A higher number of subjects might have allowed the detection of significant differences in FMD related to the subjects’ insulin sensitivity status.

## 5. Conclusions

In our study, we evaluated the efficacy of isomaltulose (Palatinose™) and sucrose loads on endothelial function as assessed by brachial ultrasound flow-mediated dilation. Our findings show that low-GI isomaltulose, compared to sucrose, led to a better preservation of the basal, pre-prandial, endothelial function in the postprandial phase. Particularly, persons with impaired insulin sensitivity seemed to benefit from the slow-release carbohydrate isomaltulose. Replacing sugar with isomaltulose may exert beneficial effects on cardiovascular health as a result of the more balanced and sustained blood glucose profile. The impact of continued isomaltulose consumption on endothelial function merits further investigations.

## Figures and Tables

**Table 1:** Baseline characteristics of the study population before the first intervention day (n = 78).^1,2^.

Gender, %	34% Females, 66% Males
Age, years	46.6 ± 8.4
Height, cm	174.4 ± 9.8
BMI, kg/m²	28.9 ± 2.6
Blood pressure (BP), mmHg	
Systolic BP	137.8 ± 5.0
Diastolic BP	88.2 ± 3.6
Fasting FMD, %	5.8 ± 2.5
Fasting blood glucose, mmol/L	4.9 ± 0.5
Fasting insulin, pmol/L	61.8 ± 28.0
HOMA index	1.9 ± 1.0
Insulin sensitivity ^3^	79% insulin sensitive, 21% insulin resistant

^1^ Values are reported as mean ± SD. ^2^ FMD, flow-mediated dilation; HOMA, homeostasis model assessment. ^3^ Classified according to the HOMA on their first intervention day: HOMA < 2.5 = insulin-sensitive; HOMA ≥ 2.5 = insulin-resistant; BMI, body mass index.

**Table 2:** Change in flow-mediated dilation from baseline in the subjects classified as insulin-sensitive and insulin-resistant according to HOMA index ^1,2^.

		FMD Change from Baseline (T0, %)			
Time Points	Insulin Sensitivity	Isomaltulose	*p* Value ^3^	Sucrose	*p* Value ^3^
**∆FMD_T1-T0_**	**IS**	−0.16 ± 2.02	0.285	−0.74 ± 1.92	0.107
	**IR**	−0.81 ± 1.96		−1.74 ± 2.54	
**∆FMD_T2-T0_**	**IS**	0.34 ± 2.33	0.180	−0.23 ± 1.92	0.435
	**IR**	−0.62 ± 2.35		−0.68 ± 2.17	
**∆FMD_T3-T0_**	**IS**	0.12 ± 2.36	0.794	0.65 ± 2.10	0.692
	**IR**	−0.07 ± 2.54		0.40 ± 2.35	

^1^ FMD, flow-mediated dilation; HOMA, homeostasis model assessment; IR, insulin resistant (HOMA ≥ 2.5); IS, insulin sensitive (HOMA < 2.5). ^2^ Values are reported as mean ± SD. ^3^ No significant differences between IR and IS subjects; unpaired *t* test: *p* > 0.05.

## References

[1] Rask-Madsen C., King G.L. (2007). Mechanisms of Disease: Endothelial dysfunction in insulin resistance and diabetes. Nat. Clin. Pract. Endocrinol. Metab..

[2] Brunner H., Cockcroft J.R., Deanfield J., Donald A., Ferrannini E., Halcox J., Kiowski W., Luscher T.F., Mancia G., Natali A. (2005). Endothelial function and dysfunction. Part II: Association with cardiovascular risk factors and diseases. A statement by the Working Group on Endothelins and Endothelial Factors of the European Society of Hypertension. J. Hypertens..

[3] Wilkins E., Wilson L., Wickramasinghe K., Bhatnagar P., Rayner M., Townsend N. (2017). European Cardiovascular Disease Statistics 2017.

[4] Wang H., Naghavi M., Allen C., Barber R.M., Bhutta Z.A., Carter A., Casey D.C., Charlson F.J., Chen A.Z., Coates M.M. (2016). Global, regional, and national life expectancy, all-cause mortality, and cause-specific mortality for 249 causes of death, 1980–2015: A systematic analysis for the Global Burden of Disease Study 2015. Lancet.

[5] Fuster V. (2014). Global burden of cardiovascular disease: Time to implement feasible strategies and to monitor results. J. Am. Coll. Cardiol..

[6] Thijssen D.H.J., Black M.A., Pyke K.E., Padilla J., Atkinson G., Harris R.A., Parker B., Widlansky M.E., Tschakovsky M.E., Green D.J. (2011). Assessment of flow-mediated dilation in humans: A methodological and physiological guideline. Am. J. Physiol. Heart Circ. Physiol..

[7] Bonetti P.O., Lerman L.O., Lerman A. (2003). Endothelial dysfunction: A marker of atherosclerotic risk. Arter. Thromb. Vasc. Biol..

[8] Manrique C., Lastra G., Sowers J.R. (2014). New insights into insulin action and resistance in the vasculature. Ann. N.Y. Acad. Sci..

[9] Muniyappa R., Sowers J.R. (2013). Role of insulin resistance in endothelial dysfunction. Rev. Endocr. Metab. Disord..

[10] Mather K.J., Mirzamohammadi B., Lteif A., Steinberg H.O., Baron A.D. (2002). Endothelin contributes to basal vascular tone and endothelial dysfunction in human obesity and type 2 diabetes. Diabetes.

[11] DeMarco V.G., Aroor A.R., Sowers J.R. (2014). The pathophysiology of hypertension in patients with obesity. Nat. Rev. Endocrinol..

[12] Bell D.S.H., O′Keefe J.H., Jellinger P. (2008). Postprandial dysmetabolism: The missing link between diabetes and cardiovascular events?. Endocr. Pract..

[13] Garber A.J. (2012). Postprandial dysmetabolism and the heart. Heart Fail. Clin..

[14] O’Keefe J.H., Bell D.S.H. (2007). Postprandial hyperglycemia/hyperlipidemia (postprandial dysmetabolism) is a cardiovascular risk factor. Am. J. Cardiol..

[15] Jovanovski E., Zurbau A., Vuksan V. (2015). Carbohydrates and endothelial function: Is a low-carbohydrate diet or a low-glycemic index diet favourable for vascular health?. Clin. Nutr. Res..

[16] Mah E., Noh S.K., Ballard K.D., Matos M.E., Volek J.S., Bruno R.S. (2011). Postprandial hyperglycemia impairs vascular endothelial function in healthy men by inducing lipid peroxidation and increasing asymmetric dimethylarginine: Arginine. J. Nutr..

[17] Watanabe K., Oba K., Suzuki T., Ouchi M., Suzuki K., Futami-Suda S., Sekimizu K., Yamamoto N., Nakano H. (2011). Oral glucose loading attenuates endothelial function in normal individual. Eur. J. Clin. Investig..

[18] Ceriello A., Assaloni R., Da Ros R., Maier A., Piconi L., Quagliaro L., Esposito K., Giugliano D. (2005). Effect of atorvastatin and irbesartan, alone and in combination, on postprandial endothelial dysfunction, oxidative stress, and inflammation in type 2 diabetic patients. Circulation.

[19] Title L.M., Cummings P.M., Giddens K., Nassar B.A. (2000). Oral glucose loading acutely attenuates endothelium-dependent vasodilation in healthy adults without diabetes: An effect prevented by vitamins C and E. J. Am. Coll. Cardiol..

[20] Shimabukuro M., Higa M., Kinjo R., Yamakawa K., Tanaka H., Kozuka C., Yabiku K., Taira S.-I., Sata M., Masuzaki H. (2014). Effects of the brown rice diet on visceral obesity and endothelial function: The BRAVO study. Br. J. Nutr..

[21] Lavi T., Karasik A., Koren-Morag N., Kanety H., Feinberg M.S., Shechter M. (2009). The acute effect of various glycemic index dietary carbohydrates on endothelial function in nondiabetic overweight and obese subjects. J. Am. Coll. Cardiol..

[22] Philippou E., Bovill-Taylor C., Rajkumar C., Vampa M.L., Ntatsaki E., Brynes A.E., Hickson M., Frost G.S. (2009). Preliminary report: The effect of a 6-month dietary glycemic index manipulation in addition to healthy eating advice and weight loss on arterial compliance and 24-hour ambulatory blood pressure in men: A pilot study. Metab. Clin. Exp..

[23] Pereira M.A., Swain J., Goldfine A.B., Rifai N., Ludwig D.S. (2004). Effects of a low-glycemic load diet on resting energy expenditure and heart disease risk factors during weight loss. JAMA.

[24] Kim D.H., Braam B. (2013). Assessment of arterial stiffness using applanation tonometry. Can. J. Physiol. Pharmacol..

[25] Liu S., Willett W.C., Stampfer M.J., Hu F.B., Franz M., Sampson L., Hennekens C.H., Manson J.E. (2000). A prospective study of dietary glycemic load, carbohydrate intake, and risk of coronary heart disease in US women. Am. J. Clin. Nutr..

[26] Mirrahimi A., de Souza R.J., Chiavaroli L., Sievenpiper J.L., Beyene J., Hanley A.J., Augustin L.S.A., Kendall C.W.C., Jenkins D.J.A. (2012). Associations of glycemic index and load with coronary heart disease events: A systematic review and meta-analysis of prospective cohorts. J. Am. Heart Assoc..

[27] Coutinho M., Gerstein H.C., Wang Y., Yusuf S. (1999). The relationship between glucose and incident cardiovascular events. A metaregression analysis of published data from 20 studies of 95,783 individuals followed for 12.4 years. Diabetes Care.

[28] Jia G., Aroor A.R., DeMarco V.G., Martinez-Lemus L.A., Meininger G.A., Sowers J.R. (2015). Vascular stiffness in insulin resistance and obesity. Front. Physiol..

[29] Ceriello A., Bortolotti N., Crescentini A., Motz E., Lizzio S., Russo A., Ezsol Z., Tonutti L., Taboga C. (1998). Antioxidant defences are reduced during the oral glucose tolerance test in normal and non-insulin-dependent diabetic subjects. Eur. J. Clin. Investig..

[30] Keller J., Kahlhöfer J., Peter A., Bosy-Westphal A. (2016). Effects of Low versus High Glycemic Index Sugar-Sweetened Beverages on Postprandial Vasodilatation and Inactivity-Induced Impairment of Glucose Metabolism in Healthy Men. Nutrients.

[31] Lina B., Jonker D., Kozianowski G. (2002). Isomaltulose (Palatinose^®^): A review of biological and toxicological studies. Food Chem. Toxicol..

[32] Atkinson F.S., Foster-Powell K., Brand-Miller J.C. (2008). International tables of glycemic index and glycemic load values: 2008. Diabetes Care.

[33] Maeda A., Miyagawa J.-I., Miuchi M., Nagai E., Konishi K., Matsuo T., Tokuda M., Kusunoki Y., Ochi H., Murai K. (2013). Effects of the naturally-occurring disaccharides, palatinose and sucrose, on incretin secretion in healthy non-obese subjects. J. Diabetes Investig..

[34] Charakida M., de Groot E., Loukogeorgakis S.P., Khan T., Lüscher T., Kastelein J.J., Gasser T., Deanfield J.E. (2013). Variability and reproducibility of flow-mediated dilatation in a multicentre clinical trial. Eur. Heart J..

[35] Kastelein J.J.P., Duivenvoorden R., Deanfield J., de Groot E., Jukema J.W., Kaski J.-C., Münzel T., Taddei S., Lehnert V., Burgess T. (2011). Rationale and design of dal-VESSEL: A study to assess the safety and efficacy of dalcetrapib on endothelial function using brachial artery flow-mediated vasodilatation. Curr. Med. Res. Opin..

[36] Mursu J., Virtanen J.K., Rissanen T.H., Tuomainen T.-P., Nykänen I., Laukkanen J.A., Kortelainen R., Voutilainen S. (2011). Glycemic index, glycemic load, and the risk of acute myocardial infarction in Finnish men: The Kuopio Ischaemic Heart Disease Risk Factor Study. Nutr. Metab. Cardiovasc. Dis..

[37] Hardy D.S., Hoelscher D.M., Aragaki C., Stevens J., Steffen L.M., Pankow J.S., Boerwinkle E. (2010). Association of glycemic index and glycemic load with risk of incident coronary heart disease among Whites and African Americans with and without type 2 diabetes: The Atherosclerosis Risk in Communities study. Ann. Epidemiol..

[38] Esposito K., Nappo F., Marfella R., Giugliano G., Giugliano F., Ciotola M., Quagliaro L., Ceriello A., Giugliano D. (2002). Inflammatory cytokine concentrations are acutely increased by hyperglycemia in humans: Role of oxidative stress. Circulation.

[39] Ceriello A., Motz E. (2004). Is oxidative stress the pathogenic mechanism underlying insulin resistance, diabetes, and cardiovascular disease? The common soil hypothesis revisited. Arter. Thromb. Vasc. Biol..

[40] Ceriello A., Taboga C., Tonutti L., Quagliaro L., Piconi L., Bais B., Da Ros R., Motz E. (2002). Evidence for an independent and cumulative effect of postprandial hypertriglyceridemia and hyperglycemia on endothelial dysfunction and oxidative stress generation: Effects of short- and long-term simvastatin treatment. Circulation.

[41] Tesfamariam B., Cohen R.A. (1992). Free radicals mediate endothelial cell dysfunction caused by elevated glucose. Am. J. Physiol..

[42] Ting H.H., Timimi F.K., Boles K.S., Creager S.J., Ganz P., Creager M.A. (1996). Vitamin C improves endothelium-dependent vasodilation in patients with non-insulin-dependent diabetes mellitus. J. Clin. Investig..

[43] Henry C.J., Kaur B., Quek R.Y.C., Camps S.G. (2017). A Low Glycaemic Index Diet Incorporating Isomaltulose Is Associated with Lower Glycaemic Response and Variability, and Promotes Fat Oxidation in Asians. Nutrients.

[44] Holub I., Gostner A., Theis S., Nosek L., Kudlich T., Melcher R., Scheppach W. (2010). Novel findings on the metabolic effects of the low glycaemic carbohydrate isomaltulose (Palatinose). Br. J. Nutr..

[45] Kalani M. (2008). The importance of endothelin-1 for microvascular dysfunction in diabetes. Vasc. Health Risk Manag..

[46] Keyhani-Nejad F., Kemper M., Schueler R., Pivovarova O., Rudovich N., Pfeiffer A.F.H. (2016). Effects of Palatinose and Sucrose Intake on Glucose Metabolism and Incretin Secretion in Subjects with Type 2 Diabetes. Diabetes Care.

[47] Mather K.J., Steinberg H.O., Baron A.D. (2013). Insulin resistance in the vasculature. J. Clin. Investig..

[48] Hurwitz B.E., Schneiderman N., Marks J.B., Mendez A.J., Gonzalez A., Llabre M.M., Smith S.R., Bizzotto R., Santini E., Manca M.L. (2015). Adaptation of beta-Cell and Endothelial Function to Carbohydrate Loading: Influence of Insulin Resistance. Diabetes.

[49] Ang M., Linn T. (2014). Comparison of the effects of slowly and rapidly absorbed carbohydrates on postprandial glucose metabolism in type 2 diabetes mellitus patients: A randomized trial. Am. J. Clin. Nutr..

[50] Basu A., Charkoudian N., Schrage W., Rizza R.A., Basu R., Joyner M.J. (2007). Beneficial effects of GLP-1 on endothelial function in humans: Dampening by glyburide but not by glimepiride. Am. J. Physiol. Endocrinol. Metab..

[51] Nyström T., Gutniak M.K., Zhang Q., Zhang F., Holst J.J., Ahrén B., Sjöholm A. (2004). Effects of glucagon-like peptide-1 on endothelial function in type 2 diabetes patients with stable coronary artery disease. Am. J. Physiol. Endocrinol. Metab..

